# Comparison of a Five-Year Survival and Cancer Recurrence between Laparoscopically Assisted and Open Colonic Resections due to Adenocarcinoma—A Single Centre Experience

**DOI:** 10.3390/medicina56020093

**Published:** 2020-02-24

**Authors:** Jurij Janež, Armand D Škapin

**Affiliations:** 1 Department of Abdominal Surgery, University Medical Centre Ljubljana, 1000 Ljubljana, Slovenia; 2 Faculty of Medicine, University of Ljubljana, 1104 Ljubljana, Slovenia; armand.skapin@gmail.com

**Keywords:** colon cancer, survival, recurrence, surgery, laparoscopy

## Abstract

*Background and objectives:* When resecting colon adenocarcinoma, surgeons decide between the use of laparoscopically assisted and open surgery. Laparoscopic resection is known to have short-term benefits over an open operation. However, researchers are not as unified about the long-term findings. The aim of this research is to elaborate on five-year post-operative differences in survival and cancer recurrence between these two different approaches. *Materials and methods:* 74 enrolled patients were evaluated five years after a primary operation. We collected dates of deaths of deceased patients and time after operation of possible recurrences. Carcinoma staging was done by a pathologist after operation. Blood samples were taken before surgery in order to measure tumor markers (CA19-9 and CEA). *Results:* Survival after colonic adenocarcinoma surgery did not differ between the two different surgical approaches (*p* = 0.151). Recurrence of cancer was not associated with the type of operation (*p* = 0.532). Patients with recurrence had a 37.6 times greater hazard ratio of dying (95% CI: [12.0, 118]; *p* < 0.001). Advanced age adversely affected survival: patients aged <65 and ≥65 years had a 97%, and 57% survival rate, respectively. Patients with elevated tumor markers at operation had a 19.1 greater hazard ratio of dying (95% CI: [5.16, 70.4]; *p* < 0.001). Patients with different TNM stages did not have any statistically significant differences in survival (HR_II_ = 2.49; 95% CI: [0.67, 9.30]; p_II_ = 0.173) (HR_III_ = 2.18; 95% CI: [0.58, 8.12]; p_III_ = 0.246) or recurrence (*p* = 0.097). *Conclusion:* The obtained results suggest that laparoscopic resection of colon cancer is not inferior from an oncologic point of view and results in a similar long-term survival and disease-free interval. Recurrence of carcinoma, older age at initial operation and elevated tumor markers, above a pre-set threshold at operation, were found to be independent factors of lower survival. We believe that the obtained results will be of benefit when choosing treatment for colon adenocarcinoma.

## 1. Introduction

Colon and rectal cancer are globally the third most commonly diagnosed cancer in males and the second most common in females. They are thus a major contributor to cancer morbidity and mortality worldwide [1]. For colon cancer, surgery remains the mainstay treatment. Laparoscopic colon surgery has become a widely accepted alternative to open colon surgery; not only for management of benign, but also of malignant colon diseases.

Laparoscopic surgery was found by multiple researchers to be superior to open surgery regarding short-term outcomes [2,3,4]. The short-term benefits of laparoscopic colon surgery are quicker return of bowel function, reduced postoperative morbidity rates and shorter length of hospital stay, compared to open surgery. On the other hand, studies comparing long-term outcomes between laparoscopic and open colon surgery are not as unified. Most researchers did not find any comparable differences in survival and disease-free intervals between the two groups [4,5,6]. However some researchers did [7,8]. The inconsistent studies about long-term outcomes make it an interesting topic for further research. It is important to know whether the operative approach taken for colon cancer affects the quality of the oncologic procedure.

When performing laparoscopic colon surgery, surgeons sometimes convert it to an open operation because different complications make it impossible to successfully finish the surgery laparoscopically. Conversion to open surgery is reported in up to 30% of patients enrolled in studies comparing open and laparoscopic colonic resection of cancer. The current evidence has shown that patients with converted resection of colon cancer have similar outcomes compared to patients undergoing a laparoscopic completed or open resection. Body mass index, local tumor extension and comorbidities are independent predictors of conversion [9].

Regardless of operative choice for malignant colonic disease, the same oncological principles must be maintained. The two most important factors are radical resection with negative resection margins and adequate lymph node dissection. The presence of metastatic lymph nodes in colon cancer patients is an important indicator of adverse prognosis [10]. According to current international guidelines, at least 12 lymph nodes should be retrieved and evaluated in the resected specimen for an adequate histopathological staging. Adequate lymphadenectomy and sufficient lymph node retrieval from the resected specimen are crucial for ensuring adequate staging, especially to prevent under-staging. A higher number of retrieved lymph nodes seem to be an independent prognostic factor for improved survival. In colon cancer patients with lymph node metastases or in cases of inadequately sampled number of lymph nodes, adjuvant chemotherapy is recommended [1]. Other indications for adjuvant chemotherapy are pT4 stage, poorly differentiated tumors, vascular or perineural invasion, lymphangiosis carcinomatosa, positive resection margins, patient in ileus, tumor perforation and stage III or stage IV [11].

The aim of our study was to investigate possible long-term differences in survival and recurrence after colonic resection due to adenocarcinoma between two different surgical approaches, laparoscopically assisted and open surgery. We have done that by evaluating a five-year survival and disease-free interval among these two groups of patients.

## 2. Materials and Methods

We performed a retrospective analysis of 74 patients, who were operated in a 1-year period due to colon adenocarcinoma between 1 June 2011 and 30 May 2012. This is a unique-center study. The inclusion criteria were laparoscopic or open resection of primary adenocarcinoma of the colon and a completed 5-year follow-up. We have excluded all patients who underwent an emergency operation due to tumor-related complications, and patients who had been previously diagnosed with a different malignant disease, patients with primary metastatic disease (stage IV) and patients with palliative primary operation (palliative resection, palliative intestinal bypass or ostomy). All patients were of the same race, ethnicity and geographical location. Post-operative chemotherapy was administered to patients according to indications [11].

The eligible participants were then divided into two groups: Patients who underwent open colonic surgery (OCS group) and patients who underwent laparoscopically-assisted surgery (LAS group). Patients who were suitable for analysis, and had a conversion from laparoscopic to open operation, were included in the open surgery group. The patients were operated on by fifteen different surgeons and a final decision on which surgical approach was used was individually tailored, and according to the operating surgeon’s preference. Additional factors affecting the operating choice were multiple previous abdominal operations, locally advanced tumor seen on pre-operatively imaging diagnostics and comorbidities (chronic obstructive pulmonary disease, cardiac disease, pulmonary hypertension). All surgeons who performed the operations were senior colorectal surgeons who have undergone at least 30 procedures. Patient assignment was made sporadically.

For our analysis we checked the vital status of our patients on 31 July 2017 at which time the 5-year follow-up ended. We collected data from the initial hospital stay, including demographics, staging of malignancy and value of tumor markers. Upon a 5-year follow-up, we checked patients’ vital status and possible recurrence.

All subjects gave their informed consent for inclusion before they participated in the study. The study was conducted in accordance with the Declaration of Helsinki, and the protocol was approved by Institutional Review Board and the Commission of the Slovenian Medical Ethics Committee (No: 0120-159-2017/4).

### Statistical Analysis

For data analysis, we used the statistical language R. Mean values and percentage values are reported. We used a t-test to compare the two groups in regard to age, TNM stages, number of patients with metastasized lymph nodes and average number of lymph nodes evaluated per patient. Survival curves were estimated using the Kaplan-Meier estimator and compared using the log-rank test. Cancer recurrence between the two groups was compared using a chi-square test. Cox regression models were used to analyze the effect of cancer recurrence, age at operation, elevated tumor markers at operation, different TNM stages and presence of lymph node metastases on survival. Hazard ratios were derived using Cox regression models. A chi-square test was also used to analyze the effect of different TNM stages on cancer recurrence, the effect of lymph node metastases on cancer recurrence and the effect of different TNM stages on the chosen type of operation. The level of significance for the analyses was set at 5%.

## 3. Results

In the study, we investigated a 5-year survival of seventy-four patients after colonic resection due to adenocarcinoma. Five of the patients underwent a conversion from laparoscopically-assisted to open-colonic resection, and were moved to OCS group for our analysis. In all of the five patients the conversion surgery was made at the beginning of the operation, so the laparoscopy was only explorative and the entire surgical procedure was performed as an open surgery. The reasons for conversion were laparoscopically inoperable tumor (in 2 patients) and adhesions in abdominal cavity (in 3 patients). We found no statistically significant differences between the two groups regarding age (*p* = 0.066), TNM stages (*p* = 0.437) and number of patients with metastasized lymph nodes (*p* = 0.299) using a t-test (Table 1).

Postoperative chemotherapy was administered to seventeen patients; to eight patients after open surgery (24% of all patients in OCS group) and to nine patients after laparoscopic surgery (23% of all patients in LAS group).

**Figure 1:**
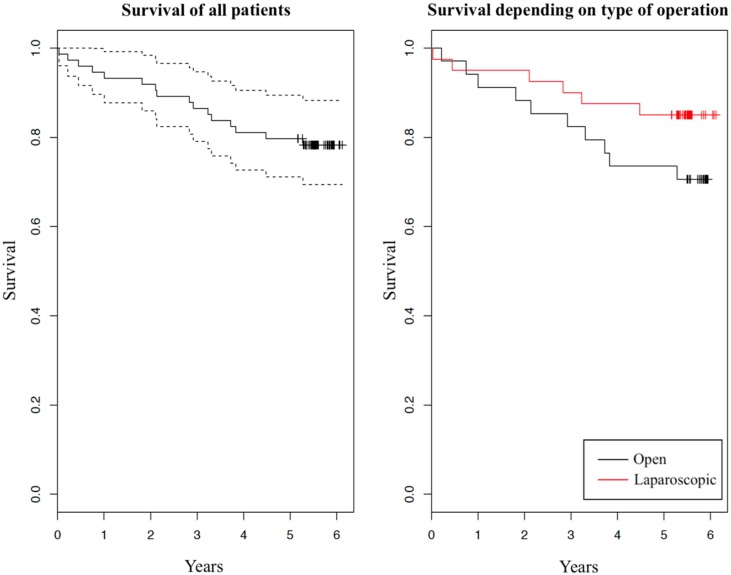
Kaplan-Meier graph of survival

Survival and carcinoma recurrence five years after the initial operation are summarized in Table 2. We can observe some difference in percentage of vital statuses between the two groups: 29% dead in OCS group versus 15% in LAS group. Taking into consideration patients’ dates of deaths, we created two graphs of survival with Kaplan–Meier estimator (Figure 1). In both graphs, survival in dependence of years after the initial operation is shown. In the left graph survival of all patients is presented (solid curve) with 95% confidence interval (dashed curves). In the right graph, survival of the two investigated groups is shown separately (red and black solid curves belong to patients after laparoscopic operation and after open operation, respectively). Comparing the tendencies of the two surgical approaches we can see a slight difference, with LAS group having a minor advantage over OCS group. We have further compared survival using the log-rank test, which showed that there is no statistically significant difference between the two groups (*p* = 0.151).

Using a chi-square test, we found recurrence of adenocarcinoma in a 5-year postoperative interval was not associated with the type of operation (*p* = 0.532). However as expected, patients with recurrence of adenocarcinoma had a 37.6 times greater hazard ratio of dying, compared to those without recurrence (95% CI: [12.0, 118]; *p* < 0.001). This was analyzed using a Cox regression model.

**Figure 2:**
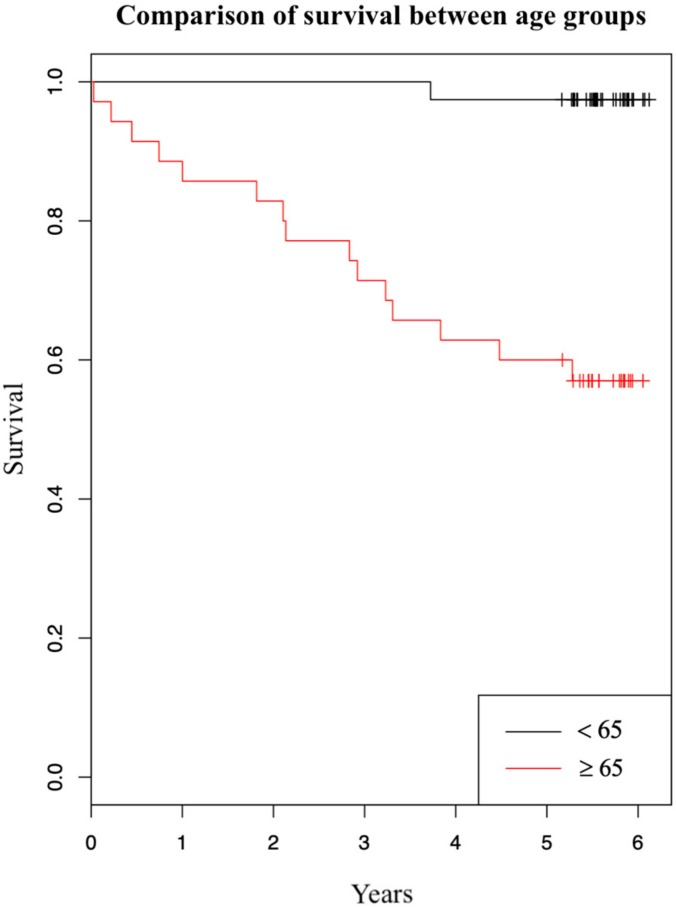
Kaplan-Meier graph of survival comparing two age groups.

We have also investigated how patients’ age at operation affects their survival. Survival five years after the initial operation by age is summarized in Table 3. We can observe a strong effect of patients’ age on survival: Patients younger than 65 years at operation had a 97% survival rate opposed to patients that were 65 years or older that had a 57% survival rate. Using Kaplan-Meier estimator we have created a graph comparing survival between the two age groups (Figure 2). In the graph survival in dependence of years after initial operation is shown. The two curves represent patients in different age groups (red and black curves belong to patients aged 65 years or older at operation and to patients younger than 65 years at operation, respectively). We have further evaluated the effect of patients’ age on survival using Cox regression analysis. It showed that one-year older patient at operation had a 13,2% greater hazard ratio of dying in five years after the initial operation (95% CI: [1.06, 1.21]; *p* < 0.001), not regarding the type of operation.

According to a Cox regression model, patients that had elevated tumor markers (CA19-9 > 30 µg/L or CEA > 4.7 µg/L) measured before the operation had a 19.1 times greater hazard ratio of dying in a 5-year time interval compared to those with measured tumor markers within normal range (95% CI: [5.16, 70.4]; *p* < 0.001).

The average number of lymph nodes evaluated was slightly higher in OCS group (16.8) than in LAS group (14.7). However, using a t-test, we did not find any statistically significant difference between the number of lymph nodes evaluated and the type of surgery (*p* = 0.167).

Using Cox regression analysis, we found patients with different TNM stages did not have any statistically significant differences in survival. More specifically, patients in the second TNM stage had a 2.49 greater hazard ratio of dying compared to those in the first TNM stage (95% CI: [0.67, 9.30]; *p* = 0.173). Similarly, patients in the third TNM stage had a 2.18 greater hazard ratio of dying compared to those in the first TNM stage (95% CI: [0.58, 8.12]; *p* = 0.246). According to a chi-square test, differences in TNM stages were not statistically significantly associated with recurrence either (*p* = 0.097). Lymph node metastases also did not affect survival (HR = 1.55; 95% CI: [0.52, 4.62]; *p* = 0.434) or recurrence (*p* = 0.064) in our patients. This was analyzed using a Cox regression analysis and a chi-square test, respectively.

TNM stage did not affect the surgeons’ choices in operative approach, which was confirmed by finding no statistically significant connection between TNM stages and the type of operation (*p* = 0.344) using a chi-square test.

## 4. Discussion

With our retrospective clinical study, we wanted to investigate the possible differences in survival and recurrence after two different surgical approaches, laparoscopically-assisted and open surgery, in patients with adenocarcinoma of the colon. Most authors agree that, there are short-term benefits of laparoscopically-assisted colonic surgery, compared with open colonic operation [2,3,4], however, we wanted to check whether there are any differences in the long-term outcome.

We found no substantial difference in five-year survival between the laparoscopically-assisted and open colonic resection due to adenocarcinoma. We did, however, observe a slightly higher percentage of survival in the LAS group versus OCS group, which can also be recognized in the graph (Figure 1). Other authors researching this topic report different results: From higher survival after laparoscopic operation [8,12], to approximately equal survival [4,6,13], to slightly higher survival after open operation [14]. Even though the percentage shows laparoscopy has a minor advantage over an open operation in our study, the difference is not statistically significant. One factor that may have contributed to this difference is the age of patients. The average age of patients was 67.0 in the OCS group and 62.7 in the LAS group. Initially, no statistically significant differences were found between the two groups in relation to age. However, age may work as a confounder [15]. Specifically, previous studies in colorectal cancer patients showed that survival is lower in patients aged 65 years or more, compared with patients younger than 65 years [16,17,18]. A stratified analysis of our study population produced a similar result (Table 3). From the table we can observe a strong effect of patients’ age on survival: patients younger than 65 years at operation had a 97% five-year survival, and patients aged 65 years or more had a 57% five-year survival. Survival difference between the two age groups is also shown in Figure 2. Additionally, we found that one-year older patient had a 13.2% greater hazard ratio of dying in five years after the operation, disregarding the type of operation. Based on Table 3, we can also see that surgeons more frequently decided to opt for laparoscopic operations in younger patients than they did in older patients, confirming age was a confounder in our study. Another factor that we found contributes to survival differences between the two surgical approaches is a higher number of unknown or other (e.g., suicide, myocardial infarction) cause of death in the OCS group (four cases in OCS group and one case in LAS group). These causes of death are not, or cannot, be linked to malignant neoplasm of colon, and this certainly does increase mortality in the OCS group. From the above findings, we can conclude that older patients in the OCS group and a higher number of deaths were not connected to malignant neoplasm in the OCS group, which surely increased the mortality in this group. Overall, the five-year survival of the enrolled patients was comparable with survival of similar patients (we excluded patients with stage IV disease) in other, larger studies researching colorectal cancer mortality [16,17].

Since the choice of the type of operation performed was up to the surgeon operating, and there were fifteen different surgeons, we were interested in whether laparoscopically-assisted operations were favored in patients with pre-operatively estimated lower primary tumor stage (assessed by abdomen CT or MRI), which could have affected the survival results. We tested that hypothesis by looking for a connection between TNM stages and the type of operation. The result was negative, meaning surgeons did not favor either type of operation, based on pre-operatively estimated primary tumor stage. However, they did favor open resection in older patients, which is unfounded since laparoscopy was found to be comparable between older and younger patients in regard to short- and long-term effects [19,20]. Nevertheless, this is a topic that could benefit from further research on a larger sample, in order to determine the true differences between the two surgical approaches on long-term effects. Due to a limited number of suitable patients in our hospital, we have enrolled only 74 patients who met the inclusion criteria. Another limitation of our study was that there was a relatively high number of surgeons with low patient/surgeon ratio. For future research we would recommend a multicenter randomized study with a larger number of patients and a relatively lower number of operating surgeons. We would also advise surgeons not to decide for either, operating technique based on patients’ age in future surgeries.

Recurrent colon adenocarcinoma is not an uncommon event, yet only a minority of patients can undergo potentially curative therapy [21]. We observed a recurrence in 16% of our patients disregarding the type of operation. The percentage was slightly higher in OCS group versus LAS group, however there was no statistically significant connection between the type of operation and recurrence. From these results we can conclude that a laparoscopically-assisted operation for colon adenocarcinoma is comparable to open operation from the oncological point of view. Other authors reported different findings; some authors found no connection between recurrence rate and type of operation [22,23]. Ringressi et al. found higher association between recurrence rate and open surgery [12]. It seems that this is another topic which would benefit from further research. Moreover, we observed that patients with recurrent adenocarcinoma had a 37.6 times greater hazard ratio of dying in a five-year time post primary operation, even though some underwent potentially curative therapy. This finding was statistically significant.

Tumor markers are measured in patients’ blood in order to evaluate the presence of carcinoma before surgery and to diagnose an early recurrence after the surgery on regular follow-ups. To evaluate the presence of colon carcinoma, we measure the amount of carcinoembryonic antigen (CEA) and carbohydrate antigen 19-9 (CA 19-9), which are found to be a good prognostic factor of survival after surgical treatment [24,25,26,27]. The survival of observed patients with measured elevated either one of the tumor markers above a pre-set threshold (CA19-9 > 30 µg/L or CEA > 4.7 µg/L) was significantly lower. To be exact, patients who had elevated tumor markers at the primary operation, had a 19.1 times greater hazard ratio of dying in a five-year time after the operation. Our findings corroborate with the finding of previous authors [24,25,26,27].

We observed no statistically significant connection between different colon carcinoma stages (evaluated with the 8th edition of the American Joint Commission on Cancer—AJCC [28]) and survival or recurrence five years after primary operation. The staging was sufficient since there were more than 12 lymph nodes collected and analyzed in most patients [1]. Other researchers have different findings regarding TNM staging system; some authors found it to be in connection with recurrence and survival [18,27], Tsikitis et al. did not find any connection and suggest that staging should include molecular characteristics of the tumor (microsatellite instability status) to be more accurate [29]. No connection between different carcinoma stages and survival could be explained by adjuvant chemotherapy, as chemotherapy was found to negate the negative prognostic factors of advanced T and N stage [29]. Another study supporting this finding, demonstrated that patients with stage IIB tumors had a higher rate of developing locoregional recurrence than patients with stage IIIA [30]. In this study only sixteen percent of patients with IIB disease received adjuvant chemotherapy, while fifty-nine percent of patients with IIIA disease received it.

Researchers’ findings about lymph nodes are even more contradictory; Qin et al. report finding lymph node metastases lowers post-operative survival [27], Mammen et al. state that with an increased number of metastasized lymph nodes, and survival also increases [31]. Qin et al. explain their findings by linking lymph node metastases to greater recurrence and distant metastases, which consequently leads to lower survival. On the other hand, Mammen et al. explain their results with more extensive lymphadenectomy and even more importantly, greater accuracy in staging leading to more sufficient adjuvant treatment. These findings could also be explained by another research study, which would find the number of evaluated lymph nodes correlates with greater survival [31,32,33], which is likewise the result of more radical operation and adjuvant chemotherapy, whenever metastasized lymph nodes are found. Current guidelines dictate that in order for an operation to be radical enough and carcinoma correctly staged, there should be a lymphadenectomy and assessment of at least 12 lymph nodes [1], if not, it can lead to under-staging, insufficient treatment and lower survival. To conclude, having locally metastasized disease does worsen survival, but finding more positive lymph nodes can be associated with more radical operations and more aggressive treatments, which improve survival. Finding no connection between survival and different carcinoma stages can, therefore, be a consequence of adequate carcinoma staging, which includes radical enough surgery and ample adjuvant therapy. Also, in observed patients there were none with IV stage, which could have influenced the results.

## 5. Conclusions

A five-year survival and disease-free interval among patients, after laparoscopic and open colonic resections, due to adenocarcinoma, was evaluated. We found no substantial difference between the two operations regarding long-term effects. We did observe a slightly higher five-year survival after laparoscopically assisted operation; however, this advantage seems to be circumstantial, since the average age of patients in the open surgery group was higher, and a higher number of deaths in the open surgery group was not connected to a malignant neoplasm. Furthermore, we found there was no difference in recurrence at the end of the five-year post-operative interval between the two groups. The drawback of our study was a somewhat small number of enrolled patients. Nevertheless, we believe that the obtained results are a good indicator, demonstrating that laparoscopic resection of colon cancer is not inferior to open resection in regard to long-term results.

The recurrence of carcinoma, older age at initial operation and elevated tumor markers above a pre-set threshold at operation, are all independent factors of lower survival.

We found no connection between patients with different carcinoma stages and survival or recurrence. This is probably the result of adequate staging, achieved by resection and assessment of at least 12 lymph nodes, and consequent sufficient adjuvant chemotherapy.

## Figures and Tables

**Table 1:** Demographic, epidemiological and pathological characteristics of enrolled patients.

Variable	Surgery Type	
	Open (*n* = 34)	Laparoscopic (*n* = 40)
Average age at operation (years)	67.0	62.7
Gender:		
Male	13 (38%)	23 (58%)
Female	21 (62%)	17 (42%)
Staging:		
0	0	0
I	12 (35%)	18 (45%)
II	11 (32%)	7 (18%)
III	8 (24%)	12 (30%)
IV	0	0
Unknown	3 (9%)	3 (7%)
Average number of evaluated lymph nodes	16.8	14.7
Lymph nodes status:		
Positive (metastasized)	8 (23%)	12 (30%)
Negative (not metastasized)	24 (71%)	27 (68%)
Unknown	2 (6%)	1 (2%)
Value of tumor markers before the operation:		
Increased (CA19-9>30 or CEA>4,7)	7 (21%)	6 (15%)
Within normal range	18 (53%)	22 (55%)
Unknown	9 (26%)	12 (30%)

**Table 2:** Vital status and recurrence of enrolled patients at a 5-year follow up.

Variable	Surgery type	
	Open (*n* = 34)	Laparoscopic (*n* = 40)
Vital status:		
Alive	24 (71%)	34 (85%)
Dead	10 (29%)	6 (15%)
Cause of death:		
Malignant neoplasm of colon	6 (60%)	5 (83%)
Other (suicide, myocardial infarction)	2 (20%)	1 (17%)
Unknown	2 (20%)	0
Recurrence of carcinoma	7 (21%)	5 (13%)

**Table 3:** Surgery type and vital status at a five-year follow up by age at operation.

	Age at Operation	
	<65 years	≥65 years
Total number of patients	39	35
Surgery type:		
Open	16 (41%)	18 (51%)
Laparoscopic	23 (59%)	17 (49%)
Vital status:		
Alive	38 (97%)	20 (57%)
Dead	1 (3%)	15 (43%)
